# *Fabp7* Maps to a Quantitative Trait Locus for a Schizophrenia Endophenotype

**DOI:** 10.1371/journal.pbio.0050297

**Published:** 2007-11-13

**Authors:** Akiko Watanabe, Tomoko Toyota, Yuji Owada, Takeshi Hayashi, Yoshimi Iwayama, Miho Matsumata, Yuichi Ishitsuka, Akihiro Nakaya, Motoko Maekawa, Tetsuo Ohnishi, Ryoichi Arai, Katsuyasu Sakurai, Kazuo Yamada, Hisatake Kondo, Kenji Hashimoto, Noriko Osumi, Takeo Yoshikawa

**Affiliations:** 1 Laboratory for Molecular Psychiatry, RIKEN Brain Science Institute, Saitama, Japan; 2 Histology, Tohoku University Graduate School of Medicine, Miyagi, Japan; 3 Animal Genome Research Unit, National Institute of Agrobiological Sciences, Ibaragi, Japan; 4 Division of Developmental Neuroscience, Center for Translational and Advanced Animal Research, Tohoku University Graduate School of Medicine, Miyagi, Japan; 5 Department of Computational Biology, University of Tokyo, Tokyo, Japan; 6 Department of Chemistry, Princeton University, Princeton, New Jersey, United States of America; 7 Division of Clinical Neuroscience, Chiba University Center for Forensic Mental Health, Chiba, Japan; 8 CREST, Japan Science and Technology Agency (JST), Saitama, Japan; North Carolina State University, United States of America

## Abstract

Deficits in prepulse inhibition (PPI) are a biological marker for schizophrenia. To unravel the mechanisms that control PPI, we performed quantitative trait loci (QTL) analysis on 1,010 F2 mice derived by crossing C57BL/6 (B6) animals that show high PPI with C3H/He (C3) animals that show low PPI. We detected six major loci for PPI, six for the acoustic startle response, and four for latency to response peak, some of which were sex-dependent. A promising candidate on the Chromosome 10-QTL was *Fabp7* (*fatty acid binding protein 7, brain*), a gene with functional links to the N-methyl-D-aspartic acid (NMDA) receptor and expression in astrocytes. *Fabp7*-deficient mice showed decreased PPI and a shortened startle response latency, typical of the QTL's proposed effects. A quantitative complementation test supported *Fabp7* as a potential PPI-QTL gene, particularly in male mice. Disruption of *Fabp7* attenuated neurogenesis in vivo*.* Human *FABP7* showed altered expression in schizophrenic brains and genetic association with schizophrenia, which were both evident in males when samples were divided by sex. These results suggest that *FABP7* plays a novel and crucial role, linking the NMDA, neurodevelopmental, and glial theories of schizophrenia pathology and the PPI endophenotype, with larger or overt effects in males. We also discuss the results from the perspective of fetal programming.

## Author Summary

A startle response to an unexpected, strong startling stimulus can be suppressed by an immediately preceding low-intensity stimulus, thereby eliciting little behavioral response. This phenomenon, called prepulse inhibition (PPI), has been observed in all mammals tested and is thought to reflect sensory-motor gating functions in organisms. PPI is diminished in human schizophrenia, raising the possibility that PPI might serve as a potential biological marker for the disease. Once the genes regulating PPI in lower animals are identified, it is expected that the human orthologs will be strong candidate genes for schizophrenia. In this study, we first performed a genetic dissection of mouse PPI using quantitative trait loci analysis, which detects chromosomal regions harboring causative genes. Further analyses including those of knockout mice, allowed us to identify one potential causative gene, *Fabp7* (*fatty acid binding protein 7, brain*), a chaperon for the essential fatty acid docosahexaenoic acid. Human studies showed that the *FABP7* gene is modestly associated with schizophrenia and that transcript expression levels are up-regulated in schizophrenic brains. From these results, we propose that a FABP7 protein-mediated disturbance of essential lipid metabolism in developing brains may be one risk factor in the development of schizophrenia, with a greater effect in males.

## Abstract 3

The search for responsible genes for prepulse inhibition, a measure deemed to be a biological trait in schizophrenia, has exposed a gene encoding essential fatty acid-binding protein.

## Introduction

Prepulse inhibition (PPI) is the normal suppression of a startle response when a low-intensity stimulus, eliciting little or no behavioral response, immediately precedes an unexpected stronger startling stimulus [1]. The response to the startling sound is called the acoustic startle reflex (ASR). PPI is observed in all mammals tested to date, and even in invertebrates [2]. As a behavioral response tool, the ASR is invaluable, because it can be measured using accessible electrophysiology tools and, importantly, measurements are possible under nearly identical conditions between humans and rodents [3]. Biologically, PPI is a reflection of sensory motor gating mechanisms within the central nervous system, and deficits within these mechanisms are a consistent feature of neuropsychiatric illnesses including schizophrenia [4]. As a reproducible phenotypic marker, impaired PPI is regarded as an endophenotype for schizophrenia. Endophenotypes are measurable internal biological markers associated with complex diseases. They provide simpler genetic clues to pathogenesis than the disease syndrome itself, allowing for easier analysis of disease [5]. It is hoped that identifying genes that underpin PPI could help in deciphering the complex polygenic mechanisms that predispose to schizophrenia.

To map the genetic loci determining PPI and its related behavioral profiles, we performed large-scale quantitative trait loci (QTL) analysis using selected inbred mouse strains. QTL analysis is a method of localizing chromosomal regions harboring genetic variants that affect a continuously distributed, polygenic phenotype [6]. Molecular dissection of the mouse genome identified *Fabp7* as a highly promising causative gene, especially in males. Functional analysis of the gene and human studies provided fresh insight into schizophrenia mechanisms and the PPI endophenotype.

## Results

### Examination of Mouse Strains for PPI Measurements

**Figure 1:**
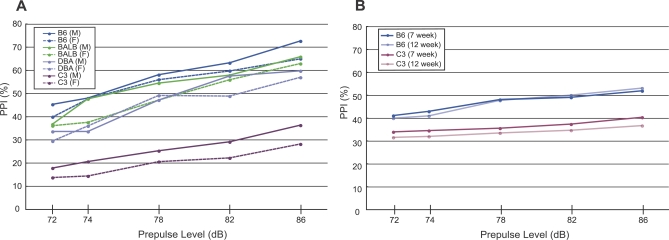
PPI (%) in Mouse Inbred Strains and at Different Ages (A) Four different strains, both males (M) and females (F) of 8–9 wk, were examined. For the results of analysis of variance, see Table S1. The comparison of male and female groups in each strain and each prepulse level did not show any significant difference by Student *t*-test. (B) Male B6 and C3 mice at 7 wk and 12 wk were examined.

In a preliminary study, we examined the supply of inbred mice for variability in the PPI response between individual animals from the same strain and consistency of PPI scores from animals with different delivery times. These longitudinal tests spanned more than 1 y. Figure 1A and Figure S1A show the PPI (%) for four different inbred mouse strains of both sexes, aged between 8–9 wk, from a specified supplier. We observed significant differences between C57BL/6 (B6) and the other strains, as well as marginally significant sex effect by analysis of variance based on the linear mixed model, in PPI magnitude (Table S1, see Materials and Methods for details). B6 mice exhibited the highest PPI and C3H/He (C3) the lowest, indicating that these two extreme strains were suitable for genetic analysis. All mouse strains and both sexes displayed increased PPI with increased prepulse levels. By testing 7-wk- and 12–wk-old B6 and C3 animals (Figure 1B and Figure S1B), we confirmed the stability of PPI during this interval. Based on these results, we used 8- to 9-wk-old cohorts for all phenotypic evaluations.

### PPI and Its Related Phenotypes in F1 and F2 Mice

We prepared a total of 1,010 F2 animals (497 males and 513 females) from F1 parents (B6 females and C3 males). For phenotypic examinations, we included measurements of ASR amplitude and latency (the interval from stimulus to maximum startle amplitude recorded in pulse-alone trials: see Figure S2), in addition to PPI at different prepulse levels. The F2 distribution of PPI values was unimodal and roughly symmetrical (skewness: 2.53 × 10^−4^ for prepulse intensity of 86 dB[A]), almost conforming to a normal distribution (kurtosis: −0.358 for prepulse intensity of 86 dB[A]) (Figure S3). The other traits also showed normal distributions (unpublished data). The mean PPI values of F0 (B6 and C3), F1, and F2 cohorts, ASR amplitudes, and latency are shown in Figure S3. We calculated broad-sense heritabilities (*h*_B_^2^) of these traits by computing the phenotypic variances (*Vp*) in the F1 [*Vp*(F1)] and F2 [*Vp*(F2)] population [*h*_B_^2^ = {*Vp*(F2) − *Vp*(F1)}/*Vp*(F2)] (Table S2): in general, these heritabilities (0.06–0.43) are low compared with those of other mouse behaviors [6] and human schizophrenia [7], which are approximately 0.5 or more. There is a tendency for PPI at higher prepulse intensities and for the male sex to give a larger heritability. The genetic correlations (*r*_A_) [8] between PPI and a range of prepulse levels, PPI and ASR, and PPI and latency are shown in Table S3. The correlation of PPI with different prepulse levels was relatively high (*r*_A_ = 0.70–0.83), which suggests that PPI is regulated by an almost identical set of genes across different prepulse intensities, an expected result. In contrast, PPI and ASR (*r*_A_ = 0.05–0.17) and PPI and latency (*r*_A_ = 0.07–0.20) correlations were low, suggesting that the genetics of ASR and latency are distinct from those of PPI. Interestingly, we found a modest correlation between ASR and latency (*r*_A_ = 0.32). Supporting these suppositions, phenotypic correlations demonstrated substantial Pearson's correlation coefficients (*r*) between PPI for different prepulse levels (*r* = 0.71–0.81) (Figure S4), whereas *r* was small between PPI and ASR (*r* = 0.04–0.16) (Figure S5) and between PPI and latency (*r* = 0.13) (Figure S5). Phenotypic correlation between ASR and latency was modest (*r* = 0.34) (Figure S5). We also confirmed that ASR was not affected by the body weight of test animals (*r* = 0.01) (Figure S5).

### First-Stage QTL Genome Scan

All 1,010 F2 generation mice were genotyped using 80 microsatellite markers covering the whole genome (19 autosomes and X chromosome) with an average intermarker distance of 20 cM [9]. QTL mapping was performed on the phenotypic and genotypic data using the method of composite interval mapping (CIM) [10], implemented in the WinQTLCart 2.5 [11] as the first stage QTL genome scan. Because the X chromosome cannot be appropriately analyzed by the WinQTLCart, we analyzed the 19 autosomes, including sex and batch, as fixed effects in the model of CIM. We classified 1,010 mice into 21 groups consisting of about 50 individuals and measured the phenotypes of mice from the same group at the same time. Accordingly, the effects of such groups were taken into consideration as the batch effects in the analysis. The results for PPI at five different prepulse levels, ASR, and latency measures are shown in Figure S6.

The mean phenotypic value for each QTL and test results for additive and dominant models are shown in Table 1. The Chromosome 10-QTL, which elicits the largest genetic influence, fits both additive and dominant models, with the former making a larger contribution than the latter. At this locus, the B6-derived allele increased PPI. Assuming that individual PPI-related QTLs act independently, genome-wide significant QTLs (Table 1) account for 26.5% of the phenotypic variation for PPI, which is comparable to the heritability of PPI (Table S2). Therefore, it is likely that the currently detected QTLs account for the almost all genetic contributions to PPI, and few if any undetected QTLs remain.

**Table 1:**
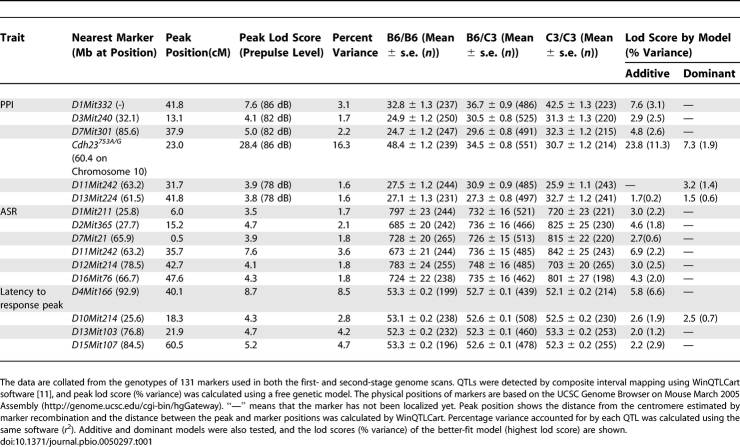
QTLs Influencing PPI and Its Related Measures

We also analyzed the X chromosome using R/qtl software [12] implemented in the statistical package R2.4.1 (http://www.r-project.org/), which can suitably handle the X chromosome in QTL analysis [13]. The results showed no significant QTL on the X chromosome. According to the analysis of variance for the phenotypes of F2 mice, the sex effect was significant in ASR (*p* < 0.01) and latency (*p* < 0.05), but not significant in PPI measures, and the batch effect was confirmed to provide no significant effects on any of the phenotypes. Therefore, neither the X chromosome nor batch effect was taken into consideration in the subsequent analyses.

### Second-Stage QTL Scanning

**Figure 2:**
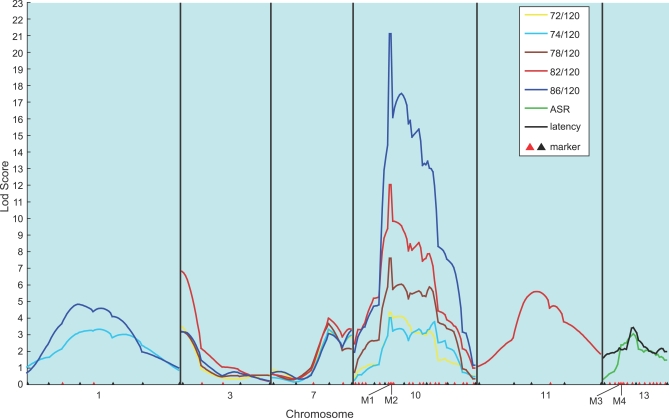
Dense Mapping in Male Mice The results from the dense mapping of chromosomes in males that harbor significant QTLs detected in the second scan are shown. The chromosomes with lod scores not exceeding the threshold are eliminated. The positions of markers used in the first scan are indicated by black triangles, and those added in the second stage scan (‘dense') by red triangles. Analysis was performed using the MIM method. M1, rs33089794, the nearest verified SNP to *Fabp7*; M2, *Cdh23* SNP; M3, *Ofcc1* SNP; M4, *Dtnbp1* SNP.

**Figure 3:**
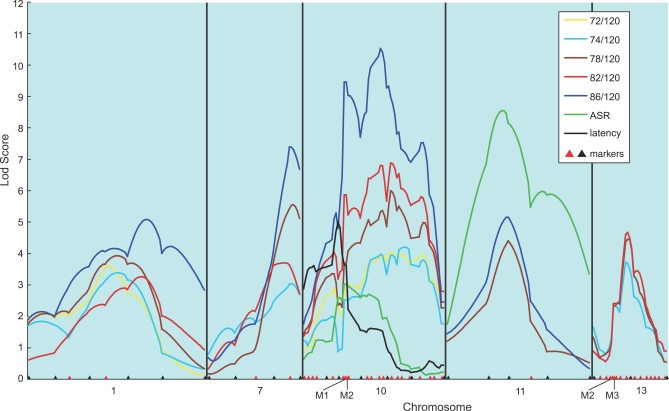
Dense Mapping in Female Mice The results from the dense mapping of chromosomes in females that harbor significant QTLs detected in the second scan are shown. The chromosomes with lod scores not exceeding the threshold are eliminated. The positions of markers used in the first scan are indicated by black triangles, and those added in the second stage scan (‘dense') by red triangles. Analysis was performed using the MIM method. M1, rs33089794; M2, *Cdh23* SNP; M3, *Ofcc1* SNP; M4, *Dtnbp1* SNP.

Our second-stage analysis involved dense mapping of the six chromosomes (1, 3, 7, 10, 11, and 13) identified at stage one that showed high CIM logarithm of the odds (lod) peaks for PPI. Additional chromosomes that are suggestive for ASR and latency were not included, because the evidence suggesting ASR and latency as endophenotypes in schizophrenia is less strong compared with PPI. We added 51 new markers, including novel single nucleotide polymorphisms (SNPs) detected in our polymorphism screen of *Cdh23* on Chromosome 10, and *Ofcc1* (*Mrds1*) and *Dtnbp1* on Chromosome 13. These also included markers from NCBI (rs33089794 near *Fabp7* locus on Chromosome 10), in addition to microsatellites from the linkage map of Dietlich et al. [9]. They covered the individual QTL intervals at a density of less than 5 cM. The positions of SNP markers on the linkage map were determined using Mapmaker/Exp 3.0 software [14]. In addition to CIM (unpublished data), we applied multiple interval mapping (MIM) [15] implemented in the WinQTLCart [11] to this second-stage analysis, where male and female mice were analyzed separately. The MIM method also allows for the epistatic interaction analysis of the QTLs [15]. We evaluated the goodness-of-model fitting in MIM with Bayesian information criterion (BIC) [16]. The model with minimized BIC can be regarded as the best model in MIM. For evaluating the significance of QTL included in the best model, we took into account the lod score for each fitted QTL, which was calculated as the twice the difference in the logarithm of likelihood between the best model and the model eliminating the QTL from the best model. We set significant lod scores for MIM, according to a genome-wide threshold of *p* < 0.05 that was calculated in the first stage using CIM. Figures 2 and 3 show the results for male and female, respectively, and they depict only the lod curves on the relevant chromosomes containing QTLs that exceeded the threshold. For PPI, Chromosome 3-QTL failed to reach the threshold in females (Figure 3). For ASR, Chromosome 11-QTL in males (Figure 2) and Chromosome 1- and 7-QTLs in females (Figure 3) were lost. For latency, the Chromosome 10-QTL in males (Figure 2) and the Chromosome 13-QTL in females (Figure 3) were also lost. The abolition of these QTLs may be related to their relatively small effect size and/or a differential sex effect. The fact that the second-stage analysis, where females and males were analyzed separately, failed to detect any chromosomes that harbor both ASR- and latency-QTLs is likely related to the observation that the two phenotypes are influenced by sex (evaluated by ANOVA, unpublished data).

Dense mapping permitted us to narrow the peak width at the Chromosome 10 PPI locus. In males, a sharp peak was observed around the *Fabp7* marker (Figure 2), and in females, one peak around the *Fabp7* locus and a second approximately 20 cM away (between *D10Mit175* and *D10Mit261*) were detected (Figure 3). (the results of a single QTL in males and potentially two QTLs in females were confirmed by performing CIM with the *Fabp7* marker (21.9 cM) as a covariate, and by analyzing male and female data separately.). In the initial scan, the Chromosome 13-QTL for PPI contained the *Ofcc1* (*Mrds1*) and the *Dtnbp1* genes in its 1-lod support interval (95% confidence interval): both genes are susceptibility candidates for human schizophrenia [17]. However, second-stage dense mapping abolished this significant PPI-QTL in males and excluded the two genes from the 1-lod support interval of the QTL in females (Figures 2 and 3). Overlapped QTLs for PPI and ASR were still evident on Chromosome 11 (females) (Figure 3) and for PPI and latency were still evident on Chromosome 10 (females) (Figure 3), suggesting that some genes may regulate multiple PPI-related features.

Epistatic interaction effects between QTLs were included in the models for some phenotypes. However, these epistases revealed no substantial effects on the phenotypes, and therefore were not analyzed further. The results obtained by using the MIM method are summarized in Table S4.

We refer to the PPI, ASR, and latency-related QTLs for combined sexes identified in this study as *Ppi-1* to *Ppi-6*, *Asr-1* to *Asr-6*, and *Latency-1* to *Latency-4*, based on the magnitude of the single-locus lod (Table 1). The data are summarized in Figure S7.

### Candidacy of *Fabp7* for *Ppi-1* and *Latency-4* on Chromosome 10

**Figure 4:**
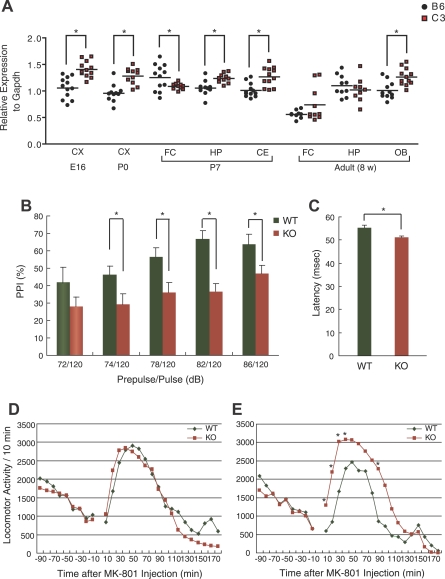
Analyses of *Fabp7* Expression and Knockout Mouse Behavior (A) Relative expression levels of *Fabp7* at different developmental stages and anatomical regions in B6 and C3 mice. The adult stage mice were all males. CX, cerebral cortex; HP, hippocampus; CE, cerebellum; FC, frontal cortex; OB, olfactory bulb, *p < 0.05 (B) PPI (%) between wild-type (WT, *n* = 9: six males and three females) and *Fabp7* (–/–) mice (KO, *n* = 12: six males and six females). The values represent mean ± s.e. *p < 0.05. (C) Latency between male wild-type (WT, *n* = 9: six males and three females) and *Fabp7* (–/–) mice (KO, *n* = 12: six males and six females). The values represent mean ± s.e. *p < 0.05. (D) Locomotor responses after a single injection of MK-801 in male wild-type (WT, *n* = 6) and *Fabp7* (–/–) mice (KO, *n* = 6). (E) Locomotor responses after a challenge injection of MK-801, following five repeated pre-treatments, in male wild-type (WT, *n* = 6) and *Fabp7* (–/–) mice (KO, *n* = 6). The genotype effect was significant [*F*(1,5) = 5.18, *p* < 0.05]. *p < 0.05 after post hoc analysis.

In the second-stage mapping, we detected a steep lod peak for PPI (at 86 dB prepulse) around *Fabp7* in males (Figure 2) and the second highest peak at the same locus in females (Figure 3). We succeeded in narrowing down genes located in the one drop lod support to approximately 30 (Ensembl release 45, June 2007, http://www.ensembl.org/Mus_musculus/index.html) in male data. In our literature survey, none of those genes (Table S5) was annotated as a modifier of PPI (this does not necessarily mean that they are true negatives). After we excluded the possible causality of *Cdh23* [18] for the QTL (see Text S1), we focused on the gene for brain-type fatty acid binding protein, *Fabp7* (also called *B-FABP*, *BLBP*) as a candidate in this QTL for the following reasons: (1) Disturbed metabolism of polyunsaturated fatty acid (PUFA), particularly *n-3* PUFA docosahexaenoic acid (DHA), has been suggested in schizophrenia [19,20,21]. (2) Fabp7 is distinguished from other fatty acid binding proteins by its strong affinity for DHA [22]. (3) Mounting evidence suggests the involvement of NMDA receptor-mediated neuronal pathways in PPI integrity and schizophrenia pathology [23], and we have obtained data underscoring the role of *Fabp7* in NMDA-mediated neural signaling (see below in this section and [24]). *Fabp7* is strongly expressed in embryonic and neonatal brains (Figures S8 and S9). In adulthood, its expression in the brain is primarily localized to astrocytes [24,25] and is markedly reduced in old mice (Figure S9). To determine whether the expression of *Fabp7* differed between the B6 and C3 lineages, we examined mRNA levels by quantitative reverse-transcriptase (RT)-PCR in the cerebral cortex of embryonic day 16 (E16) and postnatal day 0 (P0, at birth) animals; the frontal cortex, hippocampus, and cerebellum of P7 animals; and the frontal cortex, hippocampus, and olfactory bulb of adult-stage (8 wk) animals. At all developmental stages and in all anatomical regions except for P7 frontal cortex and adult frontal cortex and hippocampus, C3 animals displayed gene up-regulation compared with those from B6 (Figure 4A). In the adult frontal cortex, C3 mice showed a trend of increased expression of *Fabp7*. In contrast, C3 animals showed significantly lower expression of *Fabp7* in P7, relative to B6 animals (Figure 4A).

We hypothesized that *Fabp7* expression is strictly controlled spatio-temporally, particularly during the formation of neuronal circuitry that controls brain functions, including PPI. To test whether altered *Fabp7* expression affects PPI, we prepared *Fabp7-*deficient mice and studied their behavior. *Fabp7* (–/–) mice were viable and showed no macroscopic abnormalities [24]. Figure 4B and 4C shows that both PPI and latency were reduced in *Fabp7* (–/–) mice. The reduced latency finding was consistent with genetic analysis showing that the latency-controlling QTL was shared with the PPI-QTL on Chromosome 10 (Figure 3 and Figure S6) and C3-derived alleles at this genomic locus attenuated both PPI and latency (Table 1).

Our prior electrophysiological study using hippocampal CA1 neurons demonstrated a disruption of NMDA-induced cation current in adult *Fabp7* knockout mice [24]. Therefore, we tested whether *Fabp7* (–/–) mice showed additional features that would fit the NMDA theory of schizophrenia, by conducting pharmaco-behavioral experiments. A single challenge of a noncompetitive NMDA receptor antagonist (+)-MK-801 did not induce differences in locomotor responses between *Fabp7* (–/–) and wild-type mice (Figure 4D). But interestingly, repeated administration evoked a greater behavioral response in the *Fabp7-*deficient mice than in the wild-type counterparts (Figure 4E).

### Quantitative Complementation of *Fabp7*

Based on the candidacy of *Fabp7* as a Chromosome 10-QTL gene, we screened it for genetic polymorphisms in B6 and C3 genomic DNA. No polymorphisms were found in an interval spanning approximately 45 kb, ranging from 23 kb upstream to 18 kb downstream of the gene. The nearest annotated SNP to *Fabp7*, rs33089794 (Figures 2 and 3), is verified as being polymorphic between B6 and C3 genomes, and is located 1 Mb upstream (toward the centromere) of *Fabp7*. Quantitative genomic PCR confirmed no copy number changes to *Fabp7* in B6 and C3 mouse strains (unpublished data). All *Fabp* family genes contain a canonical TATA box followed by a conserved gene structure, but the control of tissue-specific and developmentally regulated expression of various *Fabp* types including *Fabp7* is poorly understood [26]. Therefore, we used a quantitative complementation test to determine whether *Fabp7* interacts with a Chromosome 10-QTL gene affecting PPI-phenotypes at 86 dB [27,28]. The test uses four strains: B6 and C3 that bear different QTL alleles [referred to here as high (B6) and low (C3) lines], a strain bearing a recessive mutation of *Fabp7* (m), and a wild-type strain (+) that is ideally coisogenic with the mutant. We prepared the latter two in a B6 background. We determined phenotypes of mice with the four genotypes; high/m, low/m, high/+, and low/+, and analyzed them in an experiment with two factors: “Genotype,” representing the presence or absence of the mutation at *Fabp7*, and “Line,” representing natural allelic variation between B6 and C3 at the QTL. We propose that the QTL exerts its effect by altering gene expression at a critical period and area of the brain, as might be the case if it lies in a distant enhancer element. In this case, the two effects, one due to the gene and one to the QTL, will not be independent, and their joint effect (a failure to complement) will be detected as a significant interaction between Line (high or low) and Genotype (m or +) in the analysis of variance. The interaction between Line and Genotype was significant in the combined sexes (*p* = 0.0087) and male group (*p* = 0.0232) but nonsignificant in the female group (*p* = 0.128), implicating *Fabp7* as a gene involved in the QTL with larger effects in male mice (Table 2). We did not observe significant complementation failure in latency parameter. This may be partly because of the weakness of latency-QTL on Chromosome 10 (it failed to reach the genome-wise *p* < 0.01 threshold in the first genome scan).

**Table 2:**
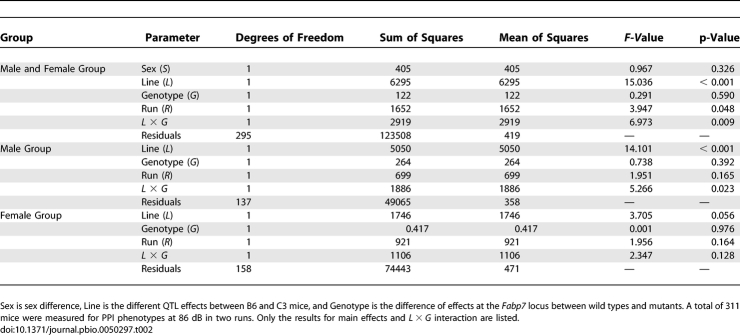
Analysis of Variance for Quantitative Complementation of the *Fabp7* Mutant

### *Fabp7*, Neurogenesis, and PPI

To gain further insight into mechanisms that could link *Fabp7* to PPI integrity and schizophrenia pathology, we examined the effects of *Fabp7* disruption on neurogenesis (defined as the proliferation of neural stem cells to produce new neurons). The rationale being that the reduction of PPI and manifestation of other schizophrenia-like behaviors correlate with a decrease of neurogenesis precipitated by the deletion of neuronal PAS domain protein 3 transcription factor [29]. As we have previously shown, *Fabp7* is essential for maintaining embryonic neural stem/progenitor cells in the developing rat cortex [30]. In addition, the expression of *Fabp7* is regulated by Pax6 [30], one of the key transcription factors in postnatal neurogenesis [31,32].

**Figure 5:**
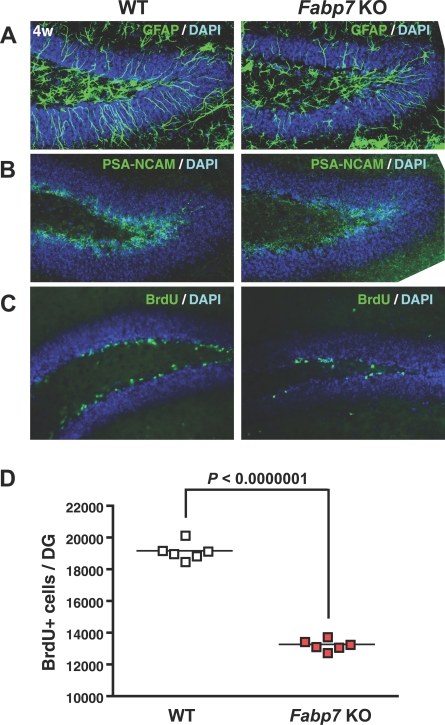
Neurogenesis in Wild-Type (WT) and *Fabp7* Knockout (KO) Mice (A and B) In the hippocampal dentate gyrus at 4 wk, *Fabp7*-null mice show decreased expression of GFAP (a neural stem/early progenitor marker) and PSA-NCAM (a late progenitor marker). (C and D) The number of BrdU-incorporated cells is markedly reduced in *Fabp7*-null mice at 4 wk, suggesting a dramatic decrease in the production of new neurons.

We performed immunostaining on the hippocampal dentate gyrus of *Fabp7* (–/–) mice at 4 wk and found a dramatic decrease in the number of GFAP (glial fibrillary acidic protein)–positive cells including astrocytes, neural stem cells, and early progenitor cells. We also noted a decrease in PSA-NCAM (a polysialylated form of the neural cell adhesion molecule) positivity, a marker for late progenitors (Figure 5A and 5B), implying that *Fabp7* deficiency impairs the maintenance of neural stem/progenitor cells. Next, we investigated cell proliferation in the dentate gyrus at 4 wk. BrdU (5'-bromo-2′-deoxyuridine)–incorporation assays showed an approximately 30% reduction in the number of dividing cells in the hippocampus of *Fabp7* (–/–) mice (Figure 5C and 5D). These results suggest that *Fabp7* plays a significant role in neurogenesis, most likely through maintenance of neural stem/progenitor cells.

### *FABP7* and Schizophrenia

**Figure 6:**
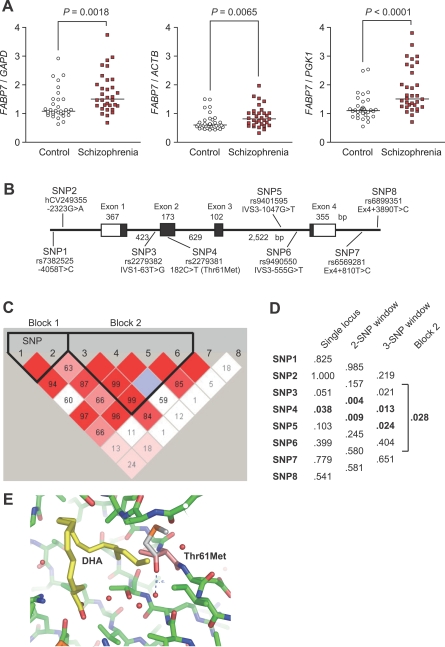
Expression and Genetic Analyses of Human *FABP7* (A) Relative mRNA levels of *FABP7* in the BA 46 region of control and schizophrenic postmortem brains. (B) Genomic structure and location of polymorphic sites in *FABP7*. Exons are denoted by boxes, with untranslated regions in white and translated regions in black. The sizes of exons and introns are also shown. “rs” is an NCBI I.D. nomenclature (http://www.ncbi.nlm.nih.gov/) and ”hCV” is derived from the Celera Discovery System (https://www.appliedbiosystems.com/). (C) The haplotype block pattern of the *FABP7* interval is shown. The number in each cell represents the linkage disequilibrium parameter *D'* (×100), red cells mean *D'* = 1. Each cell color is graduated relative to the strength of linkage disequilibrium between markers, which is defined by both the *D'* value and confidence bounds on *D'*. Note that the *FABP7* region consists of two haplotype blocks. (D) Allelic *p-*values for single-locus and global *p-*values from multilocus (two, three, and block 1) association analysis. Minor allele frequency data in control samples for individual SNPs were as follows: SNP1 (C = 0.09), SNP2 (A = 0.34), SNP3 (G = 0.36), SNP4 (T = 0.03), SNP5 (G = 0.38), SNP6 (T = 0.24), SNP7 (C = 0.10), and SNP8 (C = 0.36). (E) The site of the missense polymorphism (SNP4), Thr61Met in the FABP7 structure. The methionine residue (white) overlaps with Thr61 (magenta) of the crystal structure with DHA (yellow) (PDB ID: 1FDQ) [52]. The side-chain rotamer of methionine is arbitrary. A hydrogen bond between Thr61 and a water molecule is shown as a blue dashed line. The graphic was created using PyMOL (DeLano Scientific).

With a known association between schizophrenia and reduced PPI and a possible functional link between PPI and *Fabp7*, we examined human *FABP7* transcript levels in the postmortem brains of schizophrenics and controls. Interestingly, *FABP7* mRNA was significantly up-regulated (*p* ≤ 0.0001–0.0065, depending on the internal controls used) in the dorsolateral prefrontal cortex (Brodmann Area 46) of schizophrenics, relative to the three different internal control probes (Figure 6A). When analyses were done separately in male and female samples, the up-regulation was observed only in males (Figure S10). We propose that the up-regulation of *FABP7* is not due to the effects of neuroleptics, because chronic administration of haloperidol, a typical neuroleptics, did not enhance *Fabp7* levels in mice (Figure S11).

Next we performed a population-based genetic association study using 570 schizophrenics and 570 age/sex frequency matched controls, and analyzing eight SNPs across the gene (Figure 6B). We detected significant associations with SNP4 (*p* = 0.038), two-locus (*p* = 0.004– 0.009) and three-locus haplotypes containing SNP4 (*p* = 0.013–0.024), and haplotype block 2 (empirical *p* = 0.032 after 10,000 permutations) in which SNP4 resides (Figure 6C and 6D). It is noteworthy that SNP4 is a missense polymorphism, Thr61Met (182C>T) (rs2279381). This amino acid residue is conserved as threonine in the chimpanzee, rat, and mouse. Schizophrenic patients carried more Thr/Thr genotypes than controls (odds ratio = 1.85, 95% confidence interval = 1.06–3.24). We performed further association analysis by dividing samples according to sex. The significant association remained only in male cohorts (Figure S12). These results suggest that both expressional changes and genetic variants of *FABP7* may underlie schizophrenia pathology, with larger effects in males.

## Discussion

We have performed comprehensive QTL analyses of PPI, and other PPI-related measures, ASR and latency. Previously, only small-sized or provisional mapping studies in rodents addressed these issues [33–35]. We have also highlighted the potential molecular mechanisms of PPI and schizophrenia that are related to NMDA signaling, neurogenesis, and glial cell integrity, by analyzing a promising gene *Fabp7*.

### Genetic Effects of PPI Determinants

The current findings of small heritabilities of PPI and its related features in mice imply a strong environmental component to PPI, necessitating strict control of breeding and experimental conditions when conducting PPI experiments as a model of schizophrenia. With regard to delivery times, our experience stressed the importance of testing the reliability of the PPI phenotype at different times of arrival. Extreme developmental situations, such as animal rearing in isolation and maternal deprivation, are known to affect PPI integrity [36]. We observed a tendency for higher heritability in PPI at higher prepulse intensities, which may be relevant to the generally recognized rule in human subjects [4] and mice that stronger or more salient prepulses induce greater levels of PPI. Our results support the validity of this rule.

Joober et al. [33] estimated ASR heritability as 0.207 using congenic mice with B6 and A/J parents, and this value is very close to the 0.21 obtained in this study. To date, limited studies have focused on the characterization of latency. Zhang et al. [37] reported that a dopamine D1-like receptor agonist reduced latency but increased the startle amplitude in rats, both in a dose-dependent manner. We observed a positive, rather than negative, behavioral correlation between latency and ASR. Therefore, dopaminergic transmission provides a limited explanation for the strain differences between the two physiological measures in B6 and C3 mice.

### QTL, QTGs (Quantitative Trait Genes), and QTNs (Quantitative Trait Nucleotides)

In general, it is difficult to conclusively identify QTGs (causal genes) and even more so to identify QTNs (causal sequence variants) from QTL intervals. This is partly because a QTN could lie within a regulatory region that may be megabases away from its cognate gene [38], and there may well be multiple responsible genetic elements within a QTL and manifold epstatic gene–gene interactions for complex traits, hampering straightforward identification of QTGs/QTNs by preparing congenic crosses. We initially narrowed our research to *Fabp7*, intuitively. Then we used a complementation test to prove a role for *Fabp7* as a QTG on Chromosome 10. A significant failure to complement implies either allelism (the gene contains the functional variant) or epistasis (the gene interacts with the functional variant, which may be elsewhere in the genome). In either case, *Fabp7* is implicated as a modulator of natural variation in PPI.

### *Fabp7*, PPI, and Schizophrenia

Biological support for *Fabp7* as the sole substrate or one of a number of genetic substrates for *Ppi-1* and *Latency-4* on Chromosome 10 is demonstrated by the decrease of PPI at all levels of prepulse (although the result of the 72 dB prepulse was not significant), and the decrease of latency to startle peak seen in *Fabp7*-null mice coincides exactly with the effects of C3-derived alleles, notwithstanding the limited genetic correlation between the two traits. The down-regulation of *Fabp7* expression in the P7 frontal cortex in C3 mice compared with B6 suggests an important role for the developing frontal cortex in PPI regulation by *Fabp7*, given that *Fabp7*-defficient mice displayed disturbed PPI. *Fabp7* is expressed in various regions of the murine brain at the mid-term embryonic stage; but with progressive differentiation, *Fabp7* expression is gradually attenuated and confined to astrocytes and neural stem cells. Therefore, the role of *Fabp7* in the developing brain is predicted to be crucial. We recently showed a severe curtailing of cell proliferation in in vitro *Fabp7* knockdown experiments using small interfering RNA, but conversely an increase in neuronal differentiation [30] was shown. This study revealed an important function for *Fabp7* in neural precursor cell proliferation. Furthermore, DHA, a ligand for Fabp7, is reported to promote neurogenesis when maternally administrated [39]. These pieces of evidence suggest that the lowered expression levels of *Fabp7* detected in early postnatal stage of C3 frontal cortex might critically shift the balance from proliferation to differentiation in neuronal progenitors, thereby affecting the neuronal architecture of mature brains. The correlation between reduced neural precursor cell proliferation and impaired PPI has been shown in *Npas3-*deficient mice [40], and interestingly, the human ortholog *NPAS3* is disrupted by translocation in affected members of a family with schizophrenia [41]. Another interesting link between neurogenesis and schizophrenia is reported in the case of neuregulin1 [42–44].

It would be intriguing to speculate that up-regulation of *FABP7* in the frontal cortex of schizophrenia might be the consequence of compensatory processes for disturbed *FABP7* regulation and/or essential lipid metabolism in the developmental stages, reminiscent of “fetal programming” that is observed in metabolic syndromes [45]. It is well known that malnutrition in utero increases the probability of future schizophrenia [46,47]. *N*-3 PUFAs, in particular DHA, are considered natural ligands for FABP7, are important nutrients for nervous tissue [48], and have a special role in long-term synaptic plasticity [49]. Interestingly, our newborn *Fabp7* (–/–) mice showed a 4% decrease (*p* < 0.05) in DHA content compared to wild-type counterparts [24]. However, no significant differences were seen in the fatty acid brain content between the two groups of mice at 10 wk, suggesting the existence of compensatory mechanisms. The adult *Fabp7*-deficient mice, however, still lacked the augmentation of NMDA current in hippocampal slices that is elicited by DHA in normal mice [24], implying that the compensation is partial.

In addition to diminished PPI, the increased sensitivity of *Fabp7*-null mice to repeated administrations of MK-801 further links this gene to schizophrenia, because chronic intake of a similar NMDA antagonist, phencyclidine, induces schizophrenia-like symptoms in humans and exacerbates symptoms in chronic stabilized schizophrenic patients [50]. *FABP7* maps onto human Chromosome 6q22.31, a schizophrenia linkage region supported by a meta-analysis [51]. This study demonstrated that *FABP7* could be a novel schizophrenia susceptibility gene.

The crystal structure of FABP7, harboring Thr61 with oleic acid and DHA has been reported [52]. The missense polymorphism (Thr61Met) region is shown in Figure 6E. The replacement of Thr by Met causes an increase in hydrophobicity and breakage of a hydrogen bond releasing a water molecule. These changes may affect protein stability. In addition, since the polymorphic residue is located near the fatty acid–binding site, it is likely that the polymorphism induces an affinity change for fatty acid, which could affect functionally some biological processes, including the predisposition to schizophrenia. However, it should be emphasized that, even if the missense mutation is indeed causative in our Japanese samples, the effect of *Fabp7* on mouse PPI cannot be solely due to the same mutation, because there are no amino acid differences between B6 and C3 mice.

### *FABP7* and Sex

The current animal and human studies both point to a larger or more substantial contribution by *FABP7* to PPI and schizophrenia in males relative to females. It is well known that clinical features of schizophrenia differ between the sexes [53], including the tendency for male patients to show an earlier onset and a more severe phenotype than females. Sex differences in human brain development are also underscored [54]. In addition, fatty acid metabolism including FABP is distinct between sexes [55,56]. A database search (AliBaba2.1: http://www.gene-regulation.com/pub/programs/alibaba2/index.html) detected three and one potential estrogen receptor binding elements (EREs) in the 5′ promoter region (up to 1 kb) and 5′-untranslated region (5′-UTR) of mouse *Fabp7* gene, respectively, and one potential ERE each in the promoter region (up to 1 kb) and the 5′-UTR of human *FABP7*. However, the precise mechanisms of sex-dependent differential effects of *FABP7* on schizophrenic traits remain quite elusive.

### Conclusion

The evidence accumulated in this study consistently supports causation by *Fabp7* as the sole gene or one of the multiple genetic substrates underlying the Chromosome-10 QTL of PPI. The gene's effects may be exerted through differential regulation of transcript levels in B6 and C3 mice at a critical period. Importantly, the *FABP7* gene, which is modestly associated with schizophrenia in the current study, has the potential to link together the three compelling etiological hypotheses of schizophrenia, namely the NMDA, developmental, and glial (astrocyte) theories [57,58]. The gene appears to make a larger contribution to PPI and schizophrenia in males. The remaining newly identified but uncharacterized QTLs in this study should provide a valuable resource for continuing molecular studies into PPI and schizophrenia mechanisms. Finally, there are no established prophylactic interventions for schizophrenia. Our results raise the possibility of cohort studies to examine whether replenishment of DHA in pregnant mothers can be beneficial in reducing the chance of schizophrenia development in their offspring, especially for high-risk families. Such analyses should preferably take into account genotypes that affect function and expression of *FABP7* via both direct and indirect mechanisms.

## Materials and Methods

### Mice and PPI measurements.

The inbred strains of BALB/cAnNCrlCrlj, C3H/HeNCrlCrj (C3), C57BL/6NCrlCrlj (B6) and DBA/2NCrlCrlj, and B6C3 F1 hybrid mice (female B6 × male C3) and F2 individuals produced by randomly intercrossing the F1s were obtained from Japan's Charles River Laboratories. The animals were housed in groups of four in standard cages in a temperature and humidity-controlled room with a 12-h light/dark cycle (lights on at 08:00) and had free access to standard lab chow and tap water. All the experiments were performed between 10:00 and 14:00. The experimental procedures were approved by the RIKEN Animal Ethics Committee. The apparatus for the detection of the startle reflexes (Med Associates) consisted of six standard cages placed in sound-attenuated chambers with fan ventilation. Before each testing session, acoustic stimuli and mechanical responses were calibrated using specific devices supplied by Med Associates. A test session was composed of 49 trials, and each trial comprised prepulse sounds (0, 72, 74, 78, 82, and 86 dB[A]) pulse–(120 dB[A]) paired stimulus or a no prepulse–no pulse pair were administered (for more details, see Figure S2). Percentage PPI was calculated as {[(ASR amplitude of trial without prepulse) – (ASR amplitude of trial with prepulse)]/(ASR amplitude of trial without prepulse)} × 100.

### Effects of strain and sex on PPI with different prepulse levels: Analyses.

PPI phenotypes at each of the prepulse sounds (72, 74, 78, 82, and 86 dB) were measured for the four inbred strains (B6, C3, BALB, and DBA), where the numbers of mice measured were 30, 30, 29, and 30 for males in B6, C3, BALB, and DBA, respectively, and were 32, 32, 30, and 32 for females in B6, C3, BALB, and DBA, respectively. This experiment tested three factors: strain, sex, and prepulse level. The third factor (prepulse level) is ordinal and further was nested, since each mouse had multiple measurements. Thus, we applied the linear mixed model to the test of the difference between strains and between sexes. Statistical analysis for the linear mixed model was performed using statistical software R2.4.1 (http://www.r-project.org/) with package nlme [59]. We obtained the best model well explaining the measurements on the basis of BIC as

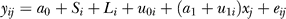
where *y_ij_* is the PPI phenotypes of the *i*th individual at the *j*th prepulse level denoted by *x_j_* equal to 72, 74, 78, 82, and 86 dB for *j* = 1, 2, 3, 4, and 5, respectively. *a*_0_ is the population average intercept, *a*_1_ is the population average slope, which is the regression coefficient of *x_j_*. *S_i_* and *L_i_* are the fixed effects of strain and sex of the *i*th individual, respectively, for which the B6 female mouse was regarded as control with *L_i_* = 0 for B6 strain and *S_i_* = 0 for females. *u*_0*i*_ and *u*_1*i*_ are the random effects of the intercept and slope associated with *i*th individual, and *e_ij_* is the within-subject error term. It is assumed that (*u*_0*i*_, *u*_1*i*_) are independently and identically distributed with a bivariate normal distribution *N*(0,σ^2^D), where D is a 2 × 2 positive definite matrix, and the *e_ij_* are independently and identically distributed with a normal distribution N(0,σ^ 2^), independent of (*u*_0*i*_, *u*_1*i*_ ). The analysis of variance showed a significant difference between B6 and the other strains as well as a marginally significant sex effect (Table S1).

We compared the PPIs of 7-wk- and 12-wk-old B6 and C3 male animals with a similar statistical method, where the effects of strains and ages were included as fixed effects in the model. As a result, a significant difference between strains was confirmed, whereas the effect of age did not differ significantly between 7- and 12-wk-old animals.

### QTL genotyping.

Microsatellite markers were typed on a 3730 *xl* DNA analyzer (Applied Biosystems) after PCR amplification using fluorescently labeled primers. For SNP genotyping, we used TaqMan probes and an ABI 7900 sequence detection system (Applied Biosystems). Detailed information on the markers used is available on request.

### QTL computation.

We used two-stage QTL analyses, the first stage using CIM [10] on the whole genome, followed by the MIM method [15] on selected chromosomes with 51 additional markers. We denote alternative alleles at a QTL originating from B6 and C3 as Q and q, respectively. In the CIM method, the following linear model was applied:

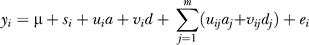
where *y_i_* is the phenotype of the *i*th individual, μ is the intercept of the model, *s_i_* is the sex effect, *u_i_* and *v_i_* are dummy variables indicating the genotype of a putative QTL on the position of interest with (*u_i_*, *v_i_*) = (1, 0), (0, 1) and (−1, 0) for QQ, Qq, and qq, respectively. *a* and *d* are additive and dominance effects of a QTL, respectively. *u_ij_* and *v_ij_* are the variables indicating the genotype of QTL near by the *j*th marker included in the model to absorb the effect of a background QTL in another region. *a_j_* and *d_j_* are the additive and dominance effects of the QTL, and *e_i_* is the residual error.

In the second QTL genome scan, we used MIM to analyze the more detailed genetic structure of the phenotypes, where male and female mice were analyzed separately. MIM uses multiple marker intervals to simultaneously construct multiple putative QTLs in the model for QTL mapping. Therefore, compared with CIM, MIM tends to be more powerful and precise in detecting QTLs. The analysis with MIM attempts to fit the following model including effects from multiple QTLs and the epistasis between QTLs to the observations:

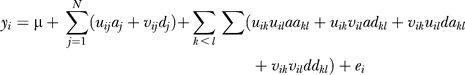
where *N* is the number of QTLs involved, *aa_kl_* is the additive by additive epistatic interaction between the *k*th QTL and the *l*th QTL, and *ad_kl_* , *da_kl_*, and *dd_kl_* are interaction of additive by dominance, dominance by additive, and dominance by dominance interaction, respectively, between the *k*th QTL and the *l*th QTL, with other parameters and variables being as described above in the model of CIM. In MIM, we evaluated the quality of model-fitting with BIC [16], which is defined as

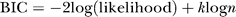
where *k* is the number of parameters fitted in the model and *n* is the sample size. The model that minimized BIC specifies the genetic architecture, namely the QTL number, *N*, the position and effect of each of *N* QTLs and epistatic interaction between some QTLs.

### Quantitative RT-PCR.

We determined the levels of mRNA from mouse and human brain tissue by real-time quantitative RT-PCR (TaqMan system and ABI 7900) and the standard curve method described elsewhere [60].

### mRNA expression data: Analyses.

The differences of the means of mRNA expression levels in mouse brains were evaluated by either Student's *t*-test (two-tailed) (two groups) or Bonferroni test (more than two groups). The gene expression levels in human brains were evaluated using the two-tailed Mann-Whitney *U*-test.

### Preparation of *Fabp7* knockout mice.

The generation of *Fabp7*-disrupted mice is described in detail elsewhere [24]. Briefly, mouse genomic clones were isolated from a 129/SvJ genomic library. The targeting vector was constructed by standard techniques. A correctly targeted ES clone was injected into the blastocyst from B6 mice. The mice used in this study were backcrossed from a mixed 129/B6 background onto B6 for at least seven generations and were intercrossed to produce wild-type and *Fabp7*-null mice.

### Behavioral responses to drug.

Adult male wild-type and *Fabp7* knockout mice (age of 8–9 wk) were used for the locomotor responses to (+)-MK-801 (Sigma Chemical). MK-801 was dissolved in physiological saline and injected intraperitoneally at a dose of 0.3 mg/kg. In chronic experiments, MK-801 was administered six times at 3-d intervals. Locomotor activities were recorded from 90 min before to 180 min after single injections (acute experiments) or after the sixth injection (chronic experiments), using Supermex (Muromati Kikai) [6].

### Locomotor activity data: Analyses.

Locomotor activity on the challenge day was analyzed using a repeated measures ANOVA (genotype × time bin) with time bin as the repeated measure, followed by Bonferroni post hoc tests.

### Quantitative complementation testing.

In the quantitative complementation test, a total of 311 mice (145 males and 166 females) were measured for PPI and latency phenotypes at two different times, where 159 mice (74 males and 85 females) and 152 mice (71 males and 81 females), respectively, were phenotyped in two independent runs. We analyzed quantitative complementation results with the statistical software R 2.4.1 using a linear model: *y* = μ + *S* + *L* + *G* + *R* + (*S* × *L*) + (*S* × *G*) + (*S* × *R*) + (*L* × *G*)+ (*L* × *R*) + (*G* × *R*) + (*S* × *L* × *G*) + (*S* × *L* × *R*) + (*S* × *G* × *R*) + (*L* × *G* × *R*) + (*S* × *L* × *G* × *R*) + *e*, for data from both males and females. Here, *y* is the phenotype; μ is the intercept; *S* is the sex effect; *L* is the difference of effects at QTL between two strains—i.e., high (B6) and low (C3);*G* is the difference of effects at a gene of interest (m and +); *R* is the effect of runs; (*S* × *L*), (*S* × *G*), (*S* × *R*), (*L* × *G*), (*L* × *R*), and (*G* × *R*) are the two-way interactions; (*S* × *L* × *G*), (*S* × *L* × *R*), (*S* × *G* × *R*), and (*L* × *G* × *R*) are the three-way interactions; (*S* × *L* × *G* × *R*) is the four-way interaction; and *e* is the residual. Moreover, we performed analyses separately for male and female mice using a model: *y* = μ + *L* + *G* + *R* + (*L* × *G*) + (*L* × *R*) + (*G* × *R*) + (*L* × *G* × *R*) + *e*. Table 2 shows only the results for main effects and *L* × *G* interaction. The detailed data are available upon request. Failure to complement is indicated by a significant interaction of (*L* × *G*) in the analysis of variance.

### Analyses of neurogenesis.

Immunostaining for NeuN (neuron specific nuclear protein), GFAP, PSA-NCAM, and BrdU was performed as previously described [30]. BrdU was injected into mice at 4 wk, three times per day for 3 d, and the brains were removed 1 d after the final injection, processed, and stained for BrdU. BrdU-incorporated cells within the whole dentate gyrus were counted as previously described [30] and statistically evaluated by Student's *t*-test (two-tailed).

### Human postmortem brain and genetic studies.

Demographic data on brain tissues (from the Stanley Medical Research Institute), and genomic DNA from patients with schizophrenia (*n* = 570) and age/sex frequency matched controls (*n* = 570), both of which were free of population stratification, have been described elsewhere [60]. We used TaqMan system for SNP genotyping. Single-locus allelic association was tested using Fisher's exact test. The haplotype block pattern of the *FABP7* interval was constructed using the Haploview program (http://www.broad.mit.edu/mpg/haploview/), using the genotype data from both case and control samples (1,140 subjects). Haplotype distributions were evaluated using the expectation-maximization algorithm implemented in the COCAPHASE of UNPHASED software package v2.403 (http://www.litbio.org). Empirical significance levels were simulated from 10,000 Monte Carlo permutations using the COCAPHASE program. The present study was approved by the ethics committee of RIKEN.

## Supporting Information

Figure S1 Bar Charts of PPI (%) in Mouse Inbred Strains and at Different Ages (A) The values represent mean ± standard error (s.e.) (*n* = 29–32 for each group). (B) The values represent mean ± s.e. (*n* = 20 for each group).
(839 KB EPS)

Figure S2 Diagram of PPI Measurements (38 KB PPT)

Figure S3 Strain Differences in PPI and Related Measures (A) Frequency histogram of PPI (%) at prepulse levels of 82 dB for each generation. The numbers of mice used for behavioral scoring were 62 for B6, 61 for C3, 210 for F1, and 1,010 for F2.
(B) PPI (%) at five different prepulse levels in each generation. The values represent mean ± s.e.
(C) Acoustic startle amplitude in each generation. The values represent mean ± s.e.
(D) Latency to startle reflex peak in each generation. The values represent mean ± s.e.
(468 KB EPS)

Figure S4 Phenotypic Correlations of PPI (%) at Different Prepulse Levels Pearson's correlation coefficients (*r*) are shown.
(144 KB PPT)

Figure S5 Phenotypic Correlations between PPI-related Measures Pearson's correlation coefficients (*r*) are shown.
(63 KB JPG)

Figure S6 Lod Score Plots for the Whole Genome The results for PPI at five different prepulse levels (72, 74, 78, 82, and 86 dB), ASR (startle stimulus was 120 dB), and latency are shown. Analysis was performed using the CIM method. Genome-wise significant threshold values were computed by permutation testing [61] of 5,000 repetitions for each trait, giving values between 3.35 and 4.23 corresponding to the genome-wise significance levels of *p* < 0.05 and 0.01 (Table S6). Peak lods exceeding the threshold for significant linkage (genome-wise *p-*value < 0.05, see [62]) were found on Chromosomes 1, 3, 7, 10, 11, and 13 for PPI on at least one prepulse sound level; on Chromosomes 1, 2, 7, 11, 12, and 16 for ASR amplitude; and on Chromosomes 4, 10, 13, and 15 for latency. PPI-QTLs on Chromosomes 1, 3, 7, and 10; ASR-QTLs on Chromosomes 2, 11, and 16; and latency-QTLs on Chromosomes 4, 13, and 15 fulfilled the criteria for highly significant linkage (genome-wise *p-*value < 0.01) (Table S6). Shared QTLs directing different PPI-related features were seen on Chromosome 10 (PPI and latency), Chromosome 11 (PPI and ASR), and Chromosome 13 (PPI and latency). Notably, the PPI-QTL on Chromosome 10 yielded the highest lod score of 27 in this study.
(87 KB JPG)

Figure S7 Chromosomal Locations of Mapped PPI, ASR, and Latency Loci Each vertical bar represents a mouse chromosome, with the centromere denoted by a black circle. Loci to the left of each chromosome are those identified in this study, and loci to the right are flanking microsatellite and SNP markers.
(382 KB EPS)

Figure S8 Expression of Fabp7 Protein in the Embryonic and Neonatal Mouse Brains Fabp7 shows abundant expression in E16 (A) and P0 (B) mouse brains. At both stages, Fabp7 is strongly expressed in the ventricular zone and radial glia. The procedure for immunohistochemistry is described elsewhere [24,32].
(42 KB JPG)

Figure S9 Northern Analysis of *Fabp7* in the Brain of Developing Mice Total RNA was extracted from whole brains of C57BL/6 mice at embryonic day 15 (E15), postnatal day 0 (P0), and postnatal day 70 (P70) by guanidine thiocyanate/phenol/chloroform extraction (triplicated for each stage). The specific 45-mer oligonucleotide probe for *Fabp7* was complementary to murine cDNA corresponding to nucleotides 6809–6853 [63]. Note that gene expression for *Fabp7* was abundant in the brain at E15 and gradually decreased at P0 and P70.
(11 KB JPG)

Figure S10 Relative mRNA Levels of *FABP7* in the BA 46 Region of Control and Schizophrenic Postmortem Brains (A) Male samples. *p*-Values were calculated using the two-tailed Mann-Whitney *U*-test. Cont, control; SZ, schizophrenia
(B) Female samples.
(493 KB EPS)

Figure S11 Effects of Chronic Haloperidol Administration on Expression Levels of *Fabp7*
Haloperidol (Dainippon Pharmaceutical, Japan; 2 mg/ml solution) was administered to C3 male mice (*n* = 12) in drinking water at concentration of 20 mg/l water for 3 wk. This dose corresponded to 1–5 mg/kg weight/day haloperidol intake, and gave serum haloperidol concentrations of 5.2–15.6 ng/ml at day 21. Control C3 male mice (*n* = 12) were given drinking water without haloperidol. On day 21, the mice were decapitated, and two brain regions (frontal cortex and hippocampus) were quickly dissected. Relative mRNA expression levels were evaluated by Student's *t*-test (two-tailed).
(29 KB PPT)

Figure S12 Association Analyses of *FABP7* in Male and Female Samples The samples consist of schizophrenia (*n* = 285 each for males and females) and age/sex-matched controls (*n* = 285 each for males and females). Single-locus allelic association was tested using Fisher's exact test. Haplotype distributions were evaluated using the expectation-maximization algorithm implemented in COCAPHASE [64]. Empirical significance levels were simulated from 10,000 Monte Carlo permutations using the COCAPHASE program.
(382 KB EPS)

Figure S13 Trial Effects on PPI and ASR in B6 and C3 Mice The values represent mean ± s.e. The number of mice used for behavioral scoring were 62 for B6 and 61 for C3.
(480 KB EPS)

Figure S14 Trial Effects on PPI and ASR in Mice with Three Different Genotypes at the *Cdh23* Locus The values represent mean ± s.e. The numbers of mice used for behavioral scoring were 156 for B6/B6, 476 for B6/C3, and 177 for C3/C3. The *Cdh23* genotypes are *Cdh23* ^753A^/*Cdh23* ^753A^ for B6/B6, *Cdh23* ^753A^/*Cdh23* ^753G^ for B6/C3, and *Cdh23* ^753G^/*Cdh23* ^753G^ for C3/C3.
(507 KB EPS)

Table S1 Analysis of Variance for the Differences in PPI Phenotypes between Strains (27 KB XLS)

Table S2 Broad Heritability of Prepulse Inhibition, Startle Reflex, and Latency (35 KB XLS)

Table S3 Genetic Correlation between PPI-related Measures (36 KB XLS)

Table S4 Summary Results Obtained Using the MIM Method (45 KB XLS)

Table S5 Genes Located within 1-lod Confidence Interval of the Chromosome 10-QTL (Male) (98 KB XLS)

Table S6 Threshold Lod Score for QTL Analysis (34 KB XLS)

Text S1 Exclusion of *Cdh23* as a Candidate Gene in PPI and ASR (38 KB DOC)

## Abbreviations

ASR: acoustic startle response
B6: C57BL/6
BIC: Bayesian information criterion
C3: C3H/He
CIM: composite interval mapping
DHA: docosahexaenoic acid
*Fabp7*: *fatty acid binding protein 7, brain*
lod: logarithm of the odds
MIM: multiple interval mapping
PPI: prepulse inhibition
PUFA: polyunsaturated fatty acid
QTL: quantitative trait loci

## References

[pbio-0050297-b001] Graham FK (1975). The more or less startling effects of weak prestimulation. Psychophysiology.

[pbio-0050297-b002] Frost WN, Tian LM, Hoppe TA, Mongeluzi DL, Wang J (2003). A cellular mechanism for prepulse inhibition. Neuron.

[pbio-0050297-b003] Swerdlow NR, Braff DL, Geyer MA (1999). Cross-species studies of sensorimotor gating of the startle reflex. Ann NY Acad Sci.

[pbio-0050297-b004] Braff DL, Geyer MA, Swerdlow NR (2001). Human studies of prepulse inhibition of startle: normal subjects, patient groups, and pharmacological studies. Psychopharmacology (Berl).

[pbio-0050297-b005] Gottesman II, Gould TD (2003). The endophenotype concept in psychiatry: etymology and strategic intentions. Am J Psychiatry.

[pbio-0050297-b006] Yoshikawa T, Watanabe A, Ishitsuka Y, Nakaya A, Nakatani N (2002). Identification of multiple genetic loci linked to the propensity for “behavioral despair” in mice. Genome Res.

[pbio-0050297-b007] Sullivan PF, Kendler K, Sakurai, Neale MC (2003). Schizophrenia as a complex trait: evidence from a meta-analysis of twin studies. Arch Gen Psychiatry.

[pbio-0050297-b008] Falconer DS, & Mackay TFC (1996). Introduction to quantitative genetics, 4th Edition.

[pbio-0050297-b009] Dietrich WF, Miller J, Steen R, Merchant MA, Damron-Boles D (1996). A comprehensive genetic map of the mouse genome. Nature.

[pbio-0050297-b010] Zen ZB (1994). Precision mapping of quantitative trait loci. Genetics.

[pbio-0050297-b011] Wang S, Basten CJ, Zeng Z-B (2007). Windows QTL Cartographer 2.5.

[pbio-0050297-b012] Broman KW, Wu H, Sen S, Churchill GA (2003). R/qtl: QTL mapping in experimental crosses. Bioinformatics.

[pbio-0050297-b013] Broman KW, Sen S, Owens SE, Manichaikul A, Southard-Smith EM (2006). The X chromosome in quantitative trait locus mapping. Genetics.

[pbio-0050297-b014] Lander ES, Green P, Abrahamson J, Barlow A, Daly MJ (1987). MAPMAKER: an interactive computer package for constructing primary genetic linkage maps of experimental and natural populations. Genomics.

[pbio-0050297-b015] Kao CH, Zeng ZB, Teasdale RD (1999). Multiple interval mapping for quantitative trait loci. Genetics.

[pbio-0050297-b016] Schwarz G (1978). Estimating the dimension of a model. Ann Stat.

[pbio-0050297-b017] Straub RE, Jiang Y, MacLean CJ, Ma Y, Webb BT (2002). Genetic variation in the 6p22.3 gene *DTNBP1*, the human ortholog of the mouse dysbindin gene, is associated with schizophrenia. Am J Hum Genet.

[pbio-0050297-b018] Noben-Trauth K, Zheng QY, Johnson KR (2003). Association of cadherin 23 with polygenic inheritance and genetic modification of sensorineural hearing loss. Nat Genet.

[pbio-0050297-b019] Pettegrew JW, Keshevan MS, Panchalingam K, Strychor S, Kaplan DB (1991). Alterations in brain high-energy phosphate and membrane phospholipid metabolism in first-episode, drug-naive schizophrenics. A pilot study of the dorsal prefrontal cortex by in vivo phosphorus 31 nuclear magnetic resonance spectrosocopy. Arch Gen Psychiatry.

[pbio-0050297-b020] Yao JK, Leonard S, Reddy RD (2000). Membrane phospolipid abnormalities in postmortem brains from schizophrenic patients. Schizophr Res.

[pbio-0050297-b021] Yao JK, Stanley JA, Reddy RD, Keshevan MS, Pettegrew JW (2002). Correlations between peripheral polyunsaturated fatty acid content and in vivo membrane phospholipid metabolites. Biol Psychiatry.

[pbio-0050297-b022] Xu LZ, Sanchez R, Sali A, Heintz N (1996). Ligand specificity of brain lipid-binding protein. J Biol Chem.

[pbio-0050297-b023] Millan MJ (2005). *N*-Methyl-d-aspartate receptors as a target for improved antipsychotic agents: novel insights and clinical perspectives. Psychopharmacology (Berl).

[pbio-0050297-b024] Owada Y, Abdelwahab SA, Kitanaka N, Sakagami H, Takano H (2006). Altered emotional behavioral responses in mice lacking brain-type fatty acid binding protein gene. Eur J Neurosci.

[pbio-0050297-b025] Owada Y, Yoshimoto T, Kondo H (1996). Spatio-temporally differential expression of genes for three members of fatty acid binding proteins in developing and mature rat brains. J Chem Neuroanat.

[pbio-0050297-b026] Haunerland NH, Spener F (2004). Fatty acid-binding proteins – insights from genetic manipulations. Prog Lipid Res.

[pbio-0050297-b027] Mackay TFC (2004). Complementing complexity. Nat Genet.

[pbio-0050297-b028] Yalcin B, Willis-Owen SAG, Fullerton J, Meesaq A, Deacon RM (2004). Genetic dissection of a behavioral quantitative trait locus shows that *Rgs2* modulates anxiety in mice. Nat Genet.

[pbio-0050297-b029] Pieper AA, Wu X, Han TW, Estill SJ, Dang Q (2005). The neuronal PAS domain protein 3 transcription factor controls FGF-mediated adult hippocampal neurogenesis in mice. Proc Natl Acad Sci U S A.

[pbio-0050297-b030] Arai Y, Funatsu N, Numayama-Tsuruta K, Nomura T, Nakamura S (2005). Role of Fabp7, a downstream gene of Pax6, in the maintenance of neuroepithelial cells during early embryonic development of the rat cortex. J Neurosci.

[pbio-0050297-b031] Hevner RF, Hodge RD, Daza R, Arai, Englund C (2006). Transcription factors in glutamatergic neurogenesis: conserved programs in neocortex, cerebellum, and adult hippocampus. Neurosci Res.

[pbio-0050297-b032] Maekawa M, Takashima N, Arai Y, Nomura T, Inokuchi K (2005). Pax6 is required for production and maintenance of progenitor cells in postnatal hippocampal neurogenesis. Genes Cells.

[pbio-0050297-b033] Joober R, Zarate JM, Roulea GA, Skamene E, Boksa P (2002). Provisional mapping of quantitative trait loci modulating the acoustic startle response and prepulse inhibition of acoustic startle. Neuropsychopharmacology.

[pbio-0050297-b034] Palmer AA, Breen LL, Flodman P, Conti LH, Spence MA (2003). Identification of quantitative trait loci for prepulse inhibition in rats. Psychopharmacology (Berl).

[pbio-0050297-b035] Petryshen TL, Kirby A, Hammer RPJr, Purcell S, O'Leary SB (2005). Two quantitative trait loci for prepulse inhibition of startle identified on mouse chromosome 16 using chromosome substitution strains. Genetics.

[pbio-0050297-b036] Geyer MA, Krebs-Thomson K, Braff DL, Swerdlow NR (2001). Pharmacological studies of prepulse inhibition models of sensorimotor gating deficits in schizophrenia: a decade in review. Psychopharmacology (Berl).

[pbio-0050297-b037] Zhang ZJ, Jiang XL, Zhang SE, Hough CJ, Li H (2005). The paradoxical effects of SKF83959, a novel dopamine D1-like receptor agonist, in the rat acoustic startle reflex paradigm. Neurosci Lett.

[pbio-0050297-b038] Lettice LA, Heaney SJ, Purdie LA, Li L, de Beer P (2003). A long-range Shh enhancer regulates expression in the developing limb and fin and is associated with preaxial polydactyly. Hum Mol Genet.

[pbio-0050297-b039] Coti Bertrand P, O'Kusky JR, Innis SM (2006). Maternal dietary (n-3) fatty acid deficiency alters neurogenesis in the embryonic rat brain. J Nutr.

[pbio-0050297-b040] Erbel-Sieler C, Dudley C, Zhou Y, Wu X, Estill SJ (2004). Behavioral and regulatory abnormalities in mice deficient in the NPAS1 and NPAS3 transcription factors. Proc Natl Acad Sci U S A.

[pbio-0050297-b041] Kamnasaran D, Muir WJ, Ferguson-Smith MA, Cox DW (2003). Disruption of the neuronal PAS3 gene in a family affected with schizophrenia. J Med Genet.

[pbio-0050297-b042] Ghashghaei HT, Weber J, Pevny L, Schmid R, Schwab MH (2006). The role of neuregulin-ErbB4 interactions on the proliferation and organization of cells in the subventricular zone. Proc Natl Acad Sci U S A.

[pbio-0050297-b043] Hahn CG, Wang HY, Cho DS, Talbot K, Gur RE (2006). Altered neuregulin 1-erbB4 signaling contributes to NMDA receptor hypofunction in schizophrenia. Nat Med.

[pbio-0050297-b044] Law AJ, Lipska BK, Weickert CS, Hyde TM, Straub RE (2006). Neuregulin 1 transcripts are differentially expressed in schizophrenia and regulated by 5' SNPs associated with the disease. Proc Natl Acad Sci U S A.

[pbio-0050297-b045] Myatt L (2006). Placental adaptive responses and fetal programming. J Physiol.

[pbio-0050297-b046] St Clair D, Xu M, Wang P, Yu Y, Fang Y (2005). Rates of adult schizophrenia following prenatal exposure to the Chinese famine of 1959–1961. JAMA.

[pbio-0050297-b047] Susser ES, Lin SP (1992). Schizophrenia after prenatal exposure to the Dutch Hunger Winter of 1944–1945. Arch Gen Psychiatry.

[pbio-0050297-b048] Fenton WS, Hibbeln J, Knable M (2000). Essential fatty acids, lipid membrane abnormalities, and the diagnosis and treatment of schizophrenia. Biol Psychiatry.

[pbio-0050297-b049] Wainwright PE (2002). Dietary essential fatty acids and brain function: a developmental perspective on mechanisms. Proc Nutr Soc.

[pbio-0050297-b050] Morris BJ, Cochran SM, Pratt JA (2005). PCP: from pharmacology to modeling schizophrenia. Curr Opin Pharmacol.

[pbio-0050297-b051] Lewis CM, Levinson DF, Wise LH, DeLisi LE, Straub RE (2003). Genome scan meta-analysis of schizophrenia and bipolar disorder, part II: schizophrenia. Am J Hum Genet.

[pbio-0050297-b052] Balendiran GK, Schnutgen F, Scapin G, Borchers T, Xhong N (2000). Crystal structure and thermodynamic analysis of human brain fatty acid-binding protein. J Biol Chem.

[pbio-0050297-b053] Nasser EH, Walders N, Jenkins JH (2002). The experience of schizophrenia: what's gender got to do with it? A critical review of the current status of research on schizophrenia. Schizophr Bull.

[pbio-0050297-b054] Goto N, Goto J (2006). Morphometric eval uations of the human nervous system. Hum Cell.

[pbio-0050297-b055] Blaak E (2001). Gender differences in fat metabolism. Curr Opin Clin Nutr Metab Care.

[pbio-0050297-b056] Luxon BA (1996). Inhibition of binding to fatty acid binding protein reduces the intracellular transport of fatty acids. Am J Physiol.

[pbio-0050297-b057] Coyle JT (2006). Glutamate and schizophrenia: beyond the dopamine hypothesis. Cell Mol Neurobiol.

[pbio-0050297-b058] Hashimoto K, Shimizu E, Iyo M (2005). Dysfunction of glia-neuron communication in pathophysiology of schizophrenia. Curr Psychiatry Rev.

[pbio-0050297-b059] Pinheiro JC, Bates M (2000). Mixed effects models in S and S-PLUS.

[pbio-0050297-b060] Shimizu H, Iwayama Y, Yamada K, Toyota T, Minabe Y (2006). Genetic and expression analyses of the ST. OhnishiP (MAP6) gene in schizophrenia. Schizophr Res.

[pbio-0050297-b061] Churchill GA, Doerge RW (1994). Empirical threshold values for quantitative trait mapping. Genetics.

[pbio-0050297-b062] Lander ES, Kruglyak L (1995). Genetic dissection of complex traits: guidelines for interpreting and reporting linkage results. Nat Genet.

[pbio-0050297-b063] Kurtz A, Zimmer A, Schnutgen F, Bruning G, Spener F (1994). The expression pattern of a novel gene encoding brain-fatty acid binding protein correlates with neuronal and glial cell development. Development.

[pbio-0050297-b064] Dudbridge F (2003). Pedigree disequilibrium tests for multilocus haplotypes. Genet Epidemiol.

[pbio-0050297-b065] Davis RR, Cheever ML, Krieg EF, Erway LC (1999). Quantitative measure of genetic differences in susceptibility to noise-induced hearing loss in two strains of mice. Hear Res.

[pbio-0050297-b066] Zheng QY, Johnson KR, Erway LC (1999). Assessment of hearing in 80 inbred strains of mice by ABR threshold analyses. Hear Res.

[pbio-0050297-b067] McCaughran J, Bell J, Hitzemann R (1999). On the relationships of high-frequency hearing loss and cochlear pathology to the acoustic startle response (ASR) and prepulse inhibition of the ASR in the BXD recombinant inbred series. Behav Genet.

[pbio-0050297-b068] Paylor R, Crawley JN (1997). Inbred strain differences in prepulse inhibition of the mouse startle response. Psychopharmacology (Berl).

