# Supplementary material for: Fabp7 Maps to a Quantitative Trait Locus for a Schizophrenia Endophenotype
Source: PLoS Biol. 2007 Nov 13;5(11):e297. doi: 10.1371/journal.pbio.0050297 (PMC2071943; doi:10.1371/journal.pbio.0050297)
Supplement: Supplementary file 1 [file pbio.0050297.sg005.pdf]

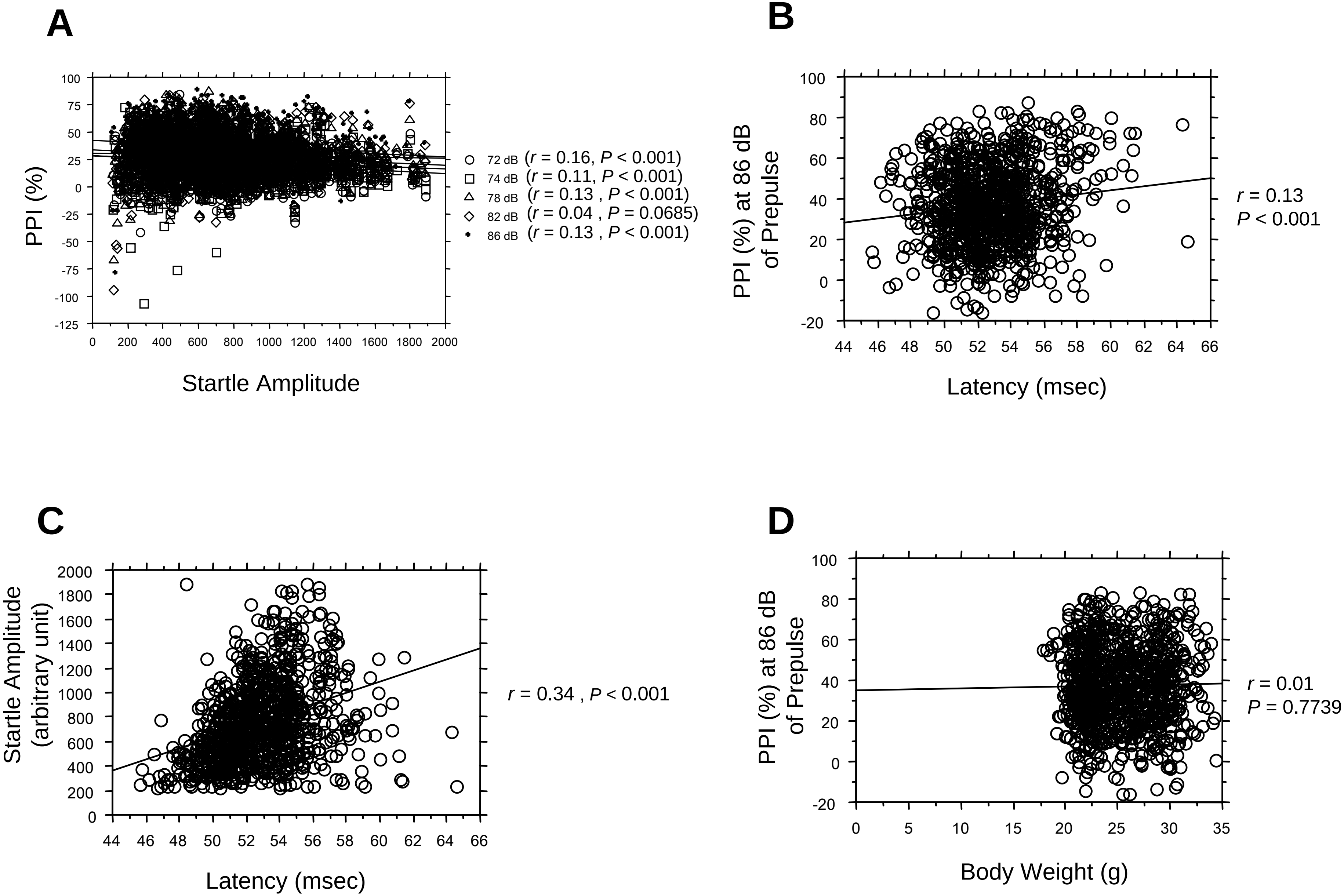

A
PPI (%)
Startle Amplitude
72 dB (r = 0.16, P < 0.001)
74 dB (r = 0.11, P < 0.001)
78 dB (r = 0.13 , P < 0.001)
82 dB (r = 0.04 , P = 0.0685)
86 dB (r = 0.13 , P < 0.001)
B
PPI (%) at 86 dB of Prepulse
Latency (msec)
r = 0.13
P < 0.001
C
Startle Amplitude (arbitrary unit)
Latency (msec)
r = 0.34 , P < 0.001
D
PPI (%) at 86 dB of Prepulse
Body Weight (g)
r = 0.01
P = 0.7739
