# Supplementary material for: Fabp7 Maps to a Quantitative Trait Locus for a Schizophrenia Endophenotype
Source: PLoS Biol. 2007 Nov 13;5(11):e297. doi: 10.1371/journal.pbio.0050297 (PMC2071943; doi:10.1371/journal.pbio.0050297)
Supplement: Supplementary file 2 [file pbio.0050297.sg006.pdf]

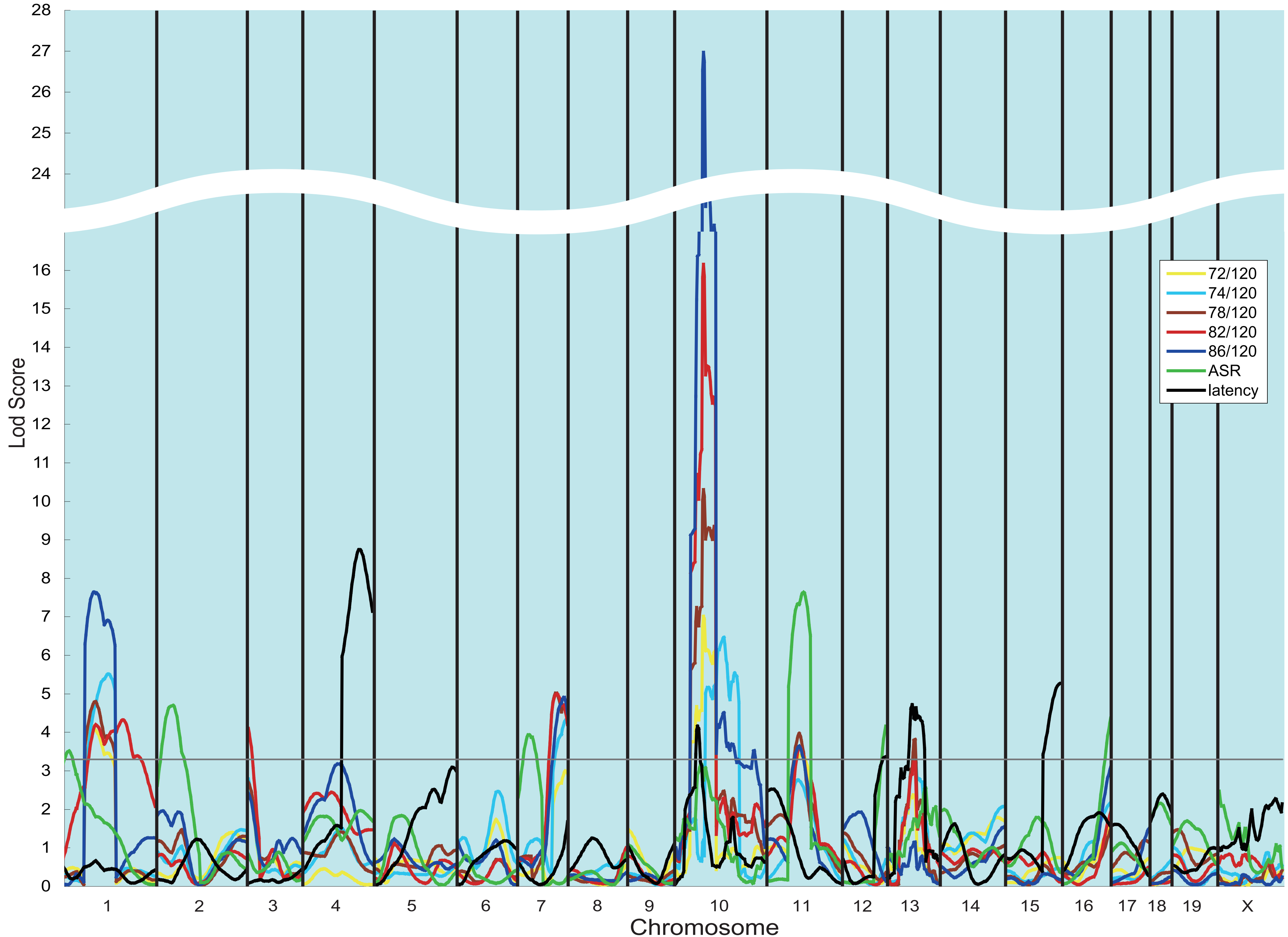

Lod Score
0
1
2
3
4
5
6
7
8
9
10
11
12
13
14
15
16
24
25
26
27
28
Chromosome
1
2
3
4
5
6
7
8
9
10
11
12
13
14
15
16
17
18
19
X
72/120
74/120
78/120
82/120
86/120
ASR
latency
