# Supplementary material for: Fabp7 Maps to a Quantitative Trait Locus for a Schizophrenia Endophenotype
Source: PLoS Biol. 2007 Nov 13;5(11):e297. doi: 10.1371/journal.pbio.0050297 (PMC2071943; doi:10.1371/journal.pbio.0050297)
Supplement: Supplementary file 3 [file pbio.0050297.sg009.pdf]

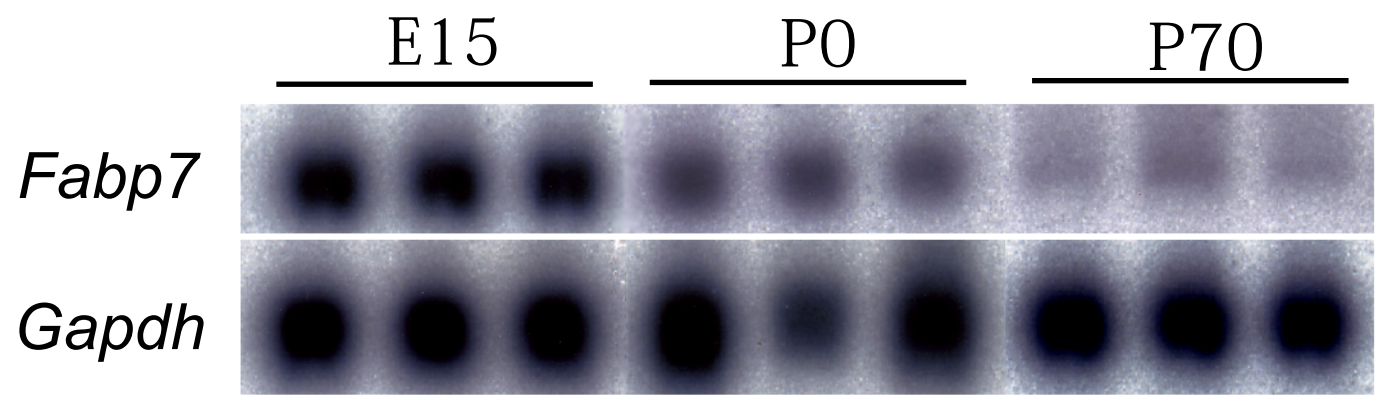
E15
P0
P70
Fabp7
Gapdh
