# Supplementary material for: Fabp7 Maps to a Quantitative Trait Locus for a Schizophrenia Endophenotype
Source: PLoS Biol. 2007 Nov 13;5(11):e297. doi: 10.1371/journal.pbio.0050297 (PMC2071943; doi:10.1371/journal.pbio.0050297)
Supplement: Supplementary file 4 [file pbio.0050297.sd001.doc]

# Text S1

**Exclusion of *Cdh23* as a candidate gene in PPI and ASR**

The present QTL on chromosome 10 spans the cadherin 23 (*Cdh23*) gene locus and homozygous *Cdh23*^753A^ (G to A transition at nucleotide 753 in exon 7; this base change causes the in-frame skipping of exon 7) alleles carried by the B6 inbred strain is reported to be associated with the noise-induced hearing loss observed in this strain [18]. Our B6 was a *Cdh23*^753A^ homozygote genotype, while the C3 was a *Cdh23*^753G^ homozygote (data not shown), and accordingly we used the polymorphism for the second stage QTL mapping as stated in the main text. We have addressed the potential of *Cdh23* as an explanation for the chromosome 10-QTL but all available evidence points to excluding *Cdh23* as a genetic variant in the QTL: we reasoned that if the *Cdh23*^753A^ mutation was responsible for the chromosome 10-QTL, the loading of trial noises in the PPI test session should have induced disturbances in PPI and the startle reflex in *Cdh23*^753A^ carrier mice. The current protocol (a session) for scoring PPI consisted of 7 trials, where in each trial 7 different prepulse-pulse paired stimuli were administered (in total 49 paired tests) against a continuous 70 dB background of white noise. We examined changes in PPI and startle reflex magnitudes during the 7 trials, with B6 and C3 strains, and observed no apparent effects on the two measures in either strain (Figure S13). We examined the above parameters in F2 progenies by dividing them according to the 753G>A genotype, and again detected no apparent trial effects in the three distinct genotype groups (Figure S14), rendering it unlikely that the genetic variant of *Cdh23* provides an explanation for the chromosome-10 QTL. In addition, “noise-induced hearing loss” is usually detectable only after administration of 110 dB noise for 4 – 8 hours [65], an incomparably large stimulus compared to those used in PPI sessions. Further evidence from literature and experiments that tended to exclude *Cdh23* as the chromosome-10 QTL included: (1) using CBA/CaJ as a reference for normal hearing, Zheng *et al* [66] reported normal auditory brainstem thresholds for B6 mice until eight months of age, (2) McCaughran *et al* [67] indicated that high frequency hearing deficits seen in many mouse strains including B6 did not affect PPI and ASR, if white noise was used as the acoustic stimuli, as was done in this study, (3) if a gene for noise-induced hearing loss was involved in a PPI-QTL, we should have observed an ASR-QTL in the same genomic region. But we did not detect a significant ASR-QTL on chromosome 10, (4) the B6 allele of *Cdh23* elicited a larger PPI and ASR (mean ± s.e.: 785 ± 29 for B6/B6, 746 ± 15 for B6/C3 and 669 ± 23 for C3/C3) than those of C3, suggesting that the current B6 has normal enough hearing to perceive prepulses and pulses, (5) *Ppi-1* acted predominantly in an additive fashion, whereas the *Cdh23*^753A^ allele induced hearing impairment only in a recessive manner [18], (6) Paylor and Crawley [68] reported mouse inbred strain differences in PPI (at prepulse of 78 dB) as follows: (high PPI to low PPI) 129/SvEvTac (*Cdh23* 753G) ~ AKR/J (*Cdh23* 753A) ~129/SvJ (*Cdh23* 753A) > FVB/NJ (*Cdh23* 753G) ~ C3H/HeJ (*Cdh23* 753G) ~ BALB/cByJ (*Cdh23* 753A) ~A/J (*Cdh23* 753A) > ST/bJ > C57BL/10J > DBA/2J (*Cdh23* 753A) > C57BL/6J (*Cdh23* 753A) (parentheses indicate the *Cdh23* 753 allele reported by Noben-Trauth *et al,* [18]). Paylor and Crawley [68] also reported inbred strain differences in ASR (with 120 dB sound) as follows: (high ASR to low ASR) C57BL/10J > FVB/NJ (*Cdh23* 753G) ~ BALB/cByJ (*Cdh23* 753A) > ST/bJ > C57BL/6J (*Cdh23* 753A) > A/J (*Cdh23* 753A) > 129/SvEvTac (*Cdh23* 753G) > AKR/J (*Cdh23* 753A) ~ C3H/HeJ (*Cdh23* 753G) > DBA/2J (*Cdh23* 753A) > 129/SvJ (*Cdh23* 753A). These results clearly indicate that the *Cdh23* 753A allele itself is neither associated with higher PPI nor higher ASR, supporting our surmise that large PPI and ASR enhancing effects elicited by the B6 chromosome 10 allele observed in this study is not attributable to *Cdh23*^753A^.
